# Streaming chunk incremental learning for class-wise data stream classification with fast learning speed and low structural complexity

**DOI:** 10.1371/journal.pone.0220624

**Published:** 2019-09-09

**Authors:** Prem Junsawang, Suphakant Phimoltares, Chidchanok Lursinsap

**Affiliations:** Department of Mathematics and Computer Science, Faculty of Science, Chulalongkorn University, Bangkok, Thailand; Politechnika Krakowska im Tadeusza Kosciuszki, POLAND

## Abstract

Due to the fast speed of data generation and collection from advanced equipment, the amount of data obviously overflows the limit of available memory space and causes difficulties achieving high learning accuracy. Several methods based on discard-after-learn concept have been proposed. Some methods were designed to cope with a single incoming datum but some were designed for a chunk of incoming data. Although the results of these approaches are rather impressive, most of them are based on temporally adding more neurons to learn new incoming data without any neuron merging process which can obviously increase the computational time and space complexities. Only online versatile elliptic basis function (VEBF) introduced neuron merging to reduce the space-time complexity of learning only a single incoming datum. This paper proposed a method for further enhancing the capability of discard-after-learn concept for streaming data-chunk environment in terms of low computational time and neural space complexities. A set of recursive functions for computing the relevant parameters of a new neuron, based on statistical confidence interval, was introduced. The newly proposed method, named streaming chunk incremental learning (SCIL), increases the plasticity and the adaptabilty of the network structure according to the distribution of incoming data and their classes. When being compared to the others in incremental-like manner, based on 11 benchmarked data sets of 150 to 581,012 samples with attributes ranging from 4 to 1,558 formed as streaming data, the proposed SCIL gave better accuracy and time in most data sets.

## 1 Introduction

Fast analysis and management of huge amounts of data by the neural computing approach is a challenging problem for current competitiveness in many research fields such as science [1–18], engineering [19, 20], medicine [21–23], social science [24–26], and business [27–30]. In contrast, the speed of data generated on the internet per unit time is tremendously faster than the possible number of bits fabricated in a very-large-scale integration (VLSI) memory chip. This contradiction creates another problem where the incoming data can overflow the memory size, which makes computation impossible. However, most of the classical learning methods require the storage of the entire training data in the memory. In this study, we consider the constraint on the data overflow and propose a new learning method based on the concept of *discard-after-learn* for temporal class-wise data chunks.

Streaming data pose challenges for machine learning, pattern recognition, and data mining. Traditional preprocess or learning approaches are not able of efficient dealing with amounts of data growing rapidly and taking into consideration characteristics, such as the distribution of the streaming data changed over time, limited computational time and memory [1]. In most machine learning applications, the streaming data are burst into a series of chunks. Each chunk may contain different data classes of various sizes. Since the data distribution is unknown and changed over time, the size of each chunk and the target of each datum are presumed to be stochastic and it could dramatically affect performance of the used model. The data stream classification has attracted extensive attention. For example, in data mining and pattern recognition, the evolving nature of data stream provides the classification difficulties in learning process and accuracy. Traditional classification techniques are usually created under complete static data given. Many learning algorithms were proposed to solve data stream classification problem, directly, such as [7–15, 17, 18]. Some stream pattern classification techniques were applied to tackle problems in real world situation. In [16], a classification method in data stream was proposed to classify patterns in the internet of things application. Jurgovsky et al. [30] applied long short-term memory (LSTM) neural network to classify transaction sequences in credit-card fraud detection problem. In addition, there are many applications that apply streaming data classifier in time series prediction, Tealab et al. [31] formulated new models of neural networks such as deep learning to predict to nonlinear times series with inherited moving average terms. Guo et al. [32] applied the adaptive learning method of LSTM network to forecast streaming time series in the presence of anomalies and change points. Mori et al. [33] employed probabilistic classifiers to early classify time series data. However, the most important challenge in this field is how to temporally and accurately classify the continuous stream of data chunks with fast computational time and limited storage units [8].

One promising solution for continuous streaming data classification is of incremental learning methods. Incremental learning algorithms can be categorized into two types based on the number of samples presented in the learning system [6], i.e. (i) online incremental learning, of which only one sample is presented for each epoch, and (ii) batch incremental learning, of which a suitable subset of samples is presented for each epoch. Polikar et al. [2] proposed an ensemble classifier for incremental learning called Learn++, in which weak hypotheses were generated and combined by weighted majority voting for class prediction. In their work, a relatively small multi-layered perceptron (MLP) acted as a based classifier or a weak classifier. Their experimental results showed that the Learn++ classifier outperformed fuzzy ARTMAP on four benchmarked and real-world data sets, but the classifier is sensitive to parameters of the network used. Wilson and Martinez [3] proposed a general inefficiency of batch learning for gradient descent learning. Based on gradient descent, their results from recognition tasks demonstrated that the incremental learning spent time less than the batch learning with no significant difference in accuracy. An incremental learning method, based the probabilistic radial basis function (PRBF) network, for classification problem in a stationary scenario was proposed [4]. The procedure of sequential component addition started with one component and repeated until any data belonged to only one class. The results of the incremental PRBF method outperformed the standard hierarchical PRBF and SVM methods in both of accuracy and computational time. Shen and Hasegawa [5] proposed a fast prototype-based nearest neighbor classifier called advanced SOINN classifier (ASC). To acquire new knowledge without losing the old one, ASC method automatically learned the suitable number of prototypes to define the decision boundary. ASC was empirically compared with other prototype-based classifiers, and the results showed that ASC provided the best performance. The limit of ASC was the difficulty of used parameters determination, and this limit cannot be applied to real-time data. All learning data must be stored for deleting a prototype with no classification usage. Duan et al. [6] presented Lagrangian support vector machines (LSVM) in both online and batch incremental algorithms. They introduced the matrix inverse computation based on previous information. The proposed LSVM was a fast and efficient algorithm compared to other online and batch incremental learning systems based on LSVM. Jaiyen, Phimoltares, and Lursinsap [7] proposed a new study based on the condition of one-pass-throw-away learning for a static environment. They also introduced the versatile elliptic basis function (VEBF) neural network using only a new incoming datum presented to the network for the learning process. This technique could be considered as a prototype-based classifier. Its technique reached the lowest bound on time complexity and achieved the smallest network structure. However, the situation of more than one datum for parameter update has not been considered. Some incremental learning algorithms cannot cope with the data stream classification, such as [5], for which the complete training data were assumed. Furthermore, many sequentially incremental learning algorithms, such as [7], were affected on the order of a presented sample or chunk of samples.

The related incremental learning methods in streaming data classification are given in Section 2. Next, Section 3 explains the studied problem. Sections 4 and 5 briefly describe the relevant background and the proposed learning concept. Section 6 presents the stream chunk incremental learning (SCIL) algorithm. The model evaluation, experimental setting, and results are discussed and given in Section 7. Finally, Section 8 concludes the paper.

## 2 Related works

Various incremental learning algorithms for streaming data classification have been widely proposed. Domingos and Hulten [9] proposed a Hoeffding tree for online learning from the high-volume data stream called the very fast decision tree. The experimental results showed its effectiveness in taking advantage of massive numbers of samples, but this method obtained a tree with quite a large size. Pang et al. [10] proposed an incremental linear discriminant analysis (ILDA), considered as incremental feature extraction in both sequential and chunk types of incoming data. The proposed ILDA was tested on various numbers of classes and features. The ILDA could effectively extract features and evolve a discriminant eigenspace to classify a fast and large data stream, when compared with the traditional LDA. Wan and Banta [11] proposed parameter incremental learning for a multi-layer perceptron (MLP) neural network. The proposed method was evaluated on both functional approximation and classification. The results showed that the speed of convergence and accuracy of the incremental MLP outperformed dramatically those of both the standard online backpropagation and the stochastic diagonal Levenberg-Marquardt (SDLM) algorithms. Ozawa et al. [12] proposed a chunk incremental principal component analysis called chunk IPCA. The discussion of chunk IPCA scalability under one-pass learning scenarios was provided. The evaluation results showed that the chunk IPCA spent less training time than the sequential IPCA to achieve the major eigenvectors. Xu et al. [13] proposed an incremental learning vector quantization (ILVQ) algorithm for pattern classification also viewed as a prototype-based classifier. The ILVQ was compared with other incremental learning methods in stationary and incremental environments. The experimental results showed that ILVQ was superior to other incremental algorithms in both of accuracy and compression ratio. Some incremental learning algorithms based on Gaussian mixture network were also proposed to handle streaming data classification with faster and scalable algorithms [14, 15]. In their works, the performance was evaluated in terms of classification accuracy, number of used components, and learning time but the performance along the course test was not evaluated as time goes by. Moreover, in some works [16, 17], data reduction techniques were applied as preprocessing step to reduce the size of training data. In [16], simple aggregation and approximation (SAX) and DB scan were used to reduce the volume of data and find the classes of incoming data. Then, support vector machine (SVM) was employed to classify the label data. The main drawback of their method is that the data were processed twice iteratively for clustering by DB scan and once for classification by SVM. In [17], a variation of random forest techniques was proposed. They applied random forest with stratified random sampling and Bloom filtering for solving steaming data classification with reducing the training time. The random sampling and Bloom filter, which were preprocessing steps, were employed to reduce the size of training samples before creating the random forest. The experiments on four types of preprocessing training data of Forest Cover Type were conducted. Their results showed that the accuracy values, measured by interleaved -test-then-train criterion, were between 0.76 and 0.88. The size of strata sampling size affected the accuracy by that the larger strata size set, the lower accuracy get.

Recently, a class-wise incremental learning (CIL) algorithm [18] was proposed to address the classification problem on large data sets. A chunk incremental learning algorithm was used to construct a versatile elliptic basis function (VEBF) network in [7]. The term “chunk incremental learning” referred to update parameters through multiple data points. Their results showed the effectiveness on high classification accuracy and the reduction in the effect of incoming datum order. However, the main drawback of their proposed technique is that the structure of a VEBF neural network may grow according to the new incoming classes and data, which makes the space complexity too high in some applications.

In this work, we propose the improvement of the learning algorithm, called SCIL, for handling streaming data. The SCIL is an expanded work of [18] to address the parameter update for multiple data points under the discard-after-learn concept, in which the data are presented to the network only once and, then, thrown away from the learning process. The proposed method protects the over-fitting problem originated from the increase of hidden neurons. The performance is expressed in terms of classification accuracy(%), the number of hidden neurons, and the computational or learning time(*s*). Moreover, the evaluation of course test on the classification of sequential incoming datum was conducted. Therefore, the VEBF network with an incremental learning algorithm for one datum is not efficient to manage the data stream scenario.

## 3 Studied problems

The aim of this work is to develop an incremental learning algorithm to handle the streaming chunk of data under the discard-after-learn concept and to make the network structure elastic and adaptive to the distribution of data chunks at different times. Generally, to learn the incoming data by neurons, there are two possible approaches. The first approach is to separate each cluster of one class from the others by using a set of neurons in forms of linear functions such as hyperplanes. The second approach is by using a set of neurons in forms of hyper-ellipsoids to capture clusters of data in the same class. Although both approaches can achieve a good performance but the first approach is not suitable for learning in a streaming data environment where the continuously incoming data can overflow the memory. To handle the memory overflow based on discard-after-learn concept, the learned data must be completely discarded and only their distribution must be captured and represented by a compact mathematical shape with a minimum set of adjustable parameters. A hyperplane cannot be used to cover the exact region of data distribution because it just separates the data space into two regions. Each region starts from the hyperplane and extends itself to infinity. Furthermore, the length and size of hyperplane of one class may interfere the data of several other classes. On the other hand, the structure of hyper-ellipsoid can cover the exact region of data distribution which makes it most suitable for the implementation of discard-after-learn concept. In this paper, the structure of hyper-ellipsoid was adopted as the structure of each neuron. A set of recursive functions for computing the relevant parameters of a new neuron, based on statistical confidence interval, was introduced. The hyper-ellipsoid is capable of translating, rotating, and scaling in all dimensions.

**Fig 1:**
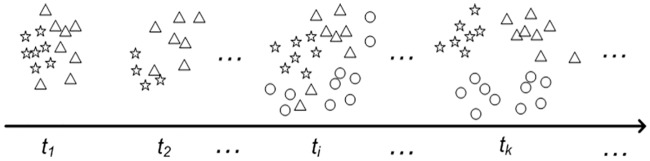
An example of streaming data with different classes of various sizes in a 2-dimensional space.

Since the incoming streaming data can contain a mixture of different classes, the following realistic scheme of class flow is concerned. Apparently, the number of classes presented in each chunk is random in real situations and unpredictable in advance before the time when a new class appears. An example of the data stream in a 2-dimensional space is shown in Fig 1. For the first data chunk at time *t*_1_, there are two classes denoted by triangles and stars. After some duration at time *t*_i_, a new class enters the learning process as denoted by circles.

In many incremental learning algorithms, the number of learning epochs is uncontrollable because the training data must be used again and again for weights update until reaching the stopping condition. Moreover, most on-line incremental learning methods have been affected by the order of presenting a datum during the learning process called the sensitivity of the learning data sequence. One of the solutions to reduce the effect of sensitivity of the sequence is learning through a data chunk with one class at a time. Once the data in any class are learned, they are completely discarded and never learned again to maintain the available memory space for the next incoming chunk. To reach the minimum number of required neurons for any class, it is essential to estimate the number of distributed sub-clusters first and to capture these sub-clusters by a set of basic activation functions to reduce the effect of misclassification. To manage the most concerned factors, which are (1) the number of uncontrollable epochs, (2) the unpredictable number of hidden neurons, and (3) the unknown prior data distribution, the following problems must be addressed.

(1) How can the number of neurons be minimized in the non-stationary streaming data with multiple classes? The goal is to achieve less neurons than those produced by the learning methods capable of coping with non-stationary streaming data.
(2) Is it possible to obtain a time complexity of *O*(*d*^2^), where *d* stands for the number of data, regardless of classes?

## 4 Relevant background

Due to the approach of the *discard-after-learn* concept, all the previously learned data cannot be recalled and mixed with those new incoming data for training the network. Hence, it is necessary to capture the region and distribution of all previously learned data of each class by a mathematical function. This function must be able to indicate the boundary of all discarded data of any classes. One of the simplest functions called VEBF introduced in [7] can be efficiently applied to this representation. In a *n*-dimensional space, we can express the shape of VEBF by

$$\begin{array}{c} \sum_{i=1}^{n}\frac{{\left((x-c\right)}^{T}u_{i}{)}^{2}}{w_{i}^{2}}\;\;=\;\;1. \end{array}$$ (1)

Set ${\left\{u_{i}\right\}}_{i=1}^{n}$ is a set of orthogonal bases, and **c** is the center. The bases **u**_*i*_ may not be the same as the original bases of the incoming-data space, but they are the bases derived from the actual direction of the data distribution. The width in dimension *i* is denoted by *w*_*i*_, and its value is estimated by using the eigenvalue of each **u**_*i*_. A data point $x\in {\mathbb{R}}^{n}$ inside or outside a VEBF can be easily determined by the following function:

$$\begin{array}{c} \psi \left(x\right)=\psi \left(x|c,U,w\right)=\sum_{i=1}^{n}\frac{{\left({\left(x-c\right)}^{T}u_{i}\right)}^{2}}{w_{i}^{2}}-1, \end{array}$$ (2)

where $U=[u_{1}\;\;u_{2}\;\;\cdots \;\;u_{n}],\;\;u_{i}\in {\mathbb{R}}^{n}$ and $w={\left[w_{1}\;\;w_{2}\;\;\cdots \;\;w_{n}\right]}^{T}\in {\mathbb{R}}^{n}$. Let $\psi_{j}^{k}(x)$ denote the VEBF of neuron *j* of class *k*. A data point $x\in {\mathbb{R}}^{n}$ is inside or covered by the *j*^*th*^ neuron of class *k* if $\psi_{j}^{k}(x)\leq 0$. Otherwise, **x** is outside neuron *j*.

During the learning process of neuron *j*, all relevant parameters are adjusted. Hence, when referring to neuron *j* of class *k* during this period, neuron *j* will be considered as a collection of these relevant parameters. Let $\Omega_{j}^{k}=(m_{j}^{k},c_{j}^{k},S_{j}^{k},U_{j}^{k},w_{j}^{k})$ be the neuron *j*^*th*^ in the subhiddel layer *k*^*th*^ with the collection of relevant parameters. The description of notations and symbols, used throughout this paper, are given in Table 1. Each class *k* contains a set of *d*_*k*_ neurons in the subhidden layer *k* (**Λ**^*k*^). The whole network is obviously formed by all sets of hidden neurons of all classes denoted as **Γ** = {**Λ**^1^, **Λ**^2^, …, **Λ**^*r*^}.

**Table 1:** The list of symbols and notations used in this paper.

Symbol	Description
**Λ**^*k*^	- *k*^*th*^ Subhidden layer, where $\Lambda^{k}=\{\Omega_{1}^{k},\Omega_{2}^{k}\ldots ,\Omega_{d_{k}}^{k}\}$.
*d*_*k*_	- Number of neurons in **Λ**^*k*^, where $d_{k}\in \mathbb{R}.$
$\Omega_{j}^{k}$	- *j*^*th*^ Neuron in **Λ**^*k*^.
$m_{j}^{k}$	- Total number of data covered by $\Omega_{j}^{k}.$
$c_{j}^{k}$	- Center vector corresponding to $\Omega_{j}^{k},$ where $c_{ji}^{k}\in {\mathbb{R}}^{n}.$
$S_{j}^{k}$	- Covariance matrix corresponding to $\Omega_{j}^{k}.$
$U_{j}^{k}$	- Matrix of orthogonal bases corresponding to $\Omega_{j}^{k}.$
$u_{j,i}^{k}$	- *i*^*th*^ Orthogonal basis of $U_{j}^{k},$ where $u_{ji}^{k}\in {\mathbb{R}}^{n}.$
$w_{j}^{k}$	- Width vector corresponding to $\Omega_{j}^{k},$ where $w_{ji}^{k}\in {\mathbb{R}}^{n}.$
$\psi_{j}^{k}(x)$	- VEBF value of a given input vector **x** at $\Omega_{j}^{k}.$

## 5 Proposed concept of Stream Chunk Incremental Learning (SCIL)

**Fig 2:**
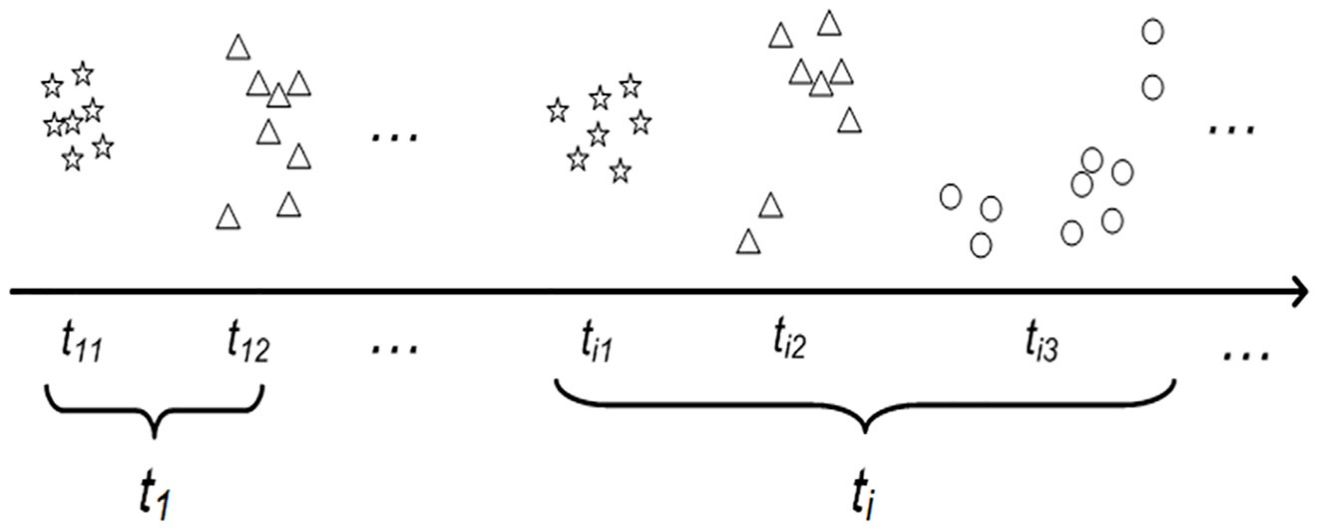
An example of class-wise data chunk.

The size of a VEBF in the original algorithm reported in [7] can be expanded. Their approach was infeasible in terms of number of neurons and learning time when handling streaming data chunk. The disadvantage was improved by gradually expanding the size of a VEBF to cover all data in the same class. CIL [18] covers one datum and expands the size of VEBF later to cover another datum in the same class if the distance of the second datum is close to the VEBF. The class-wise streaming data is used for the learning process. The example of the class-wise streaming data in a 2-dimensional space is shown in Fig 2. At any time *t*_i_, one or many chunks of data of different classes may enter the learning process. However, if the distance is too far away, then a new VEBF is introduced to cover the second datum. This approach is iterated until all data in the same class are covered. However, it is possible that two VEBFs are far apart at some past periods, but they can be near each other at the present time due to the expansion to cover new incoming data belonging to the same class. In fact, CIL does not merge these two near VEBF into one VEBF. This will increase the number of VEBFs and the computational complexity during the learning and testing processes.

For each **Λ**^*k*^, the number of neurons in any class *k* can be increased due to the large distance between an uncovered datum in that class and the existing VEBFs in **Λ**^*k*^. Too many neurons can cause the over fitting problem. Therefore, one approach to prevent the increase of neurons is to merge two near hidden neurons in the same **Λ**^*k*^. Two hidden neurons $\Omega_{\alpha }^{k}=\left(m_{\alpha }^{k},c_{\alpha }^{k},S_{\alpha }^{k},U_{\alpha }^{k},w_{\alpha }^{k}\right)$ and $\Omega_{\beta }^{k}=\left(m_{\beta }^{k},c_{\beta }^{k},S_{\beta }^{k},U_{\beta }^{k},w_{\beta }^{k}\right)$ are merged if the following condition is satisfied:

$$\begin{array}{c} \psi_{\alpha }^{k}(c_{\beta }^{k})\leq 0\;\;\;or\;\;\;\psi_{\beta }^{k}(c_{\alpha }^{k})\leq 0. \end{array}$$ (3)

**Fig 3:**
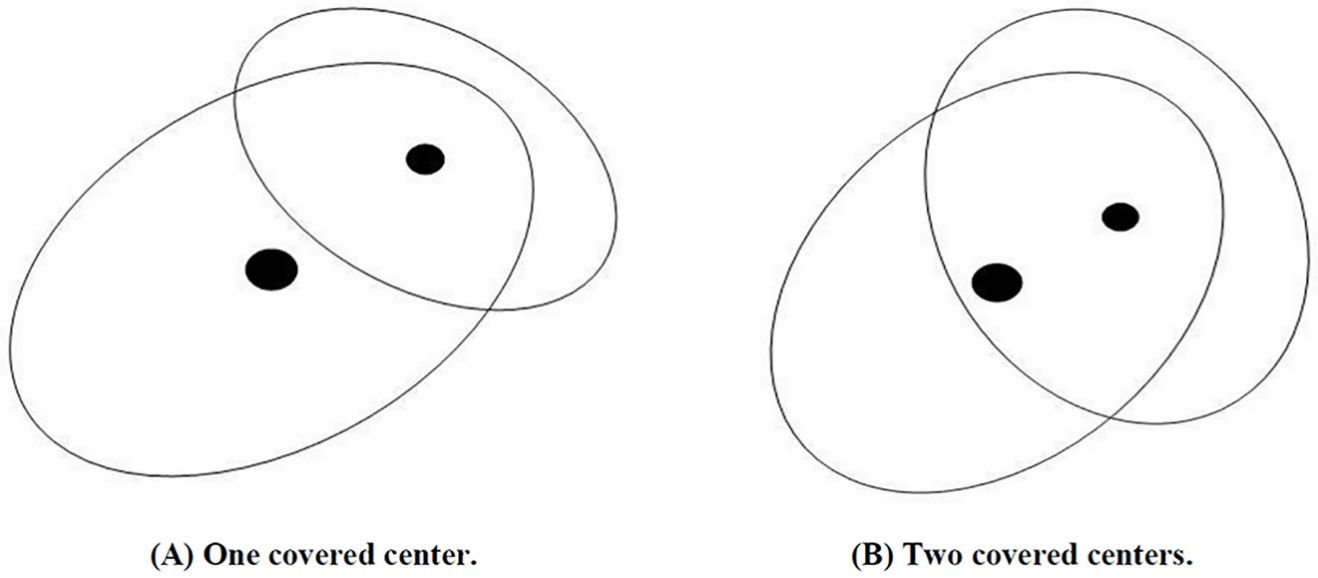
Two overlapping conditions for merging two neurons.

This means that either $\Omega_{\alpha }^{k}$ or $\Omega_{\beta }^{k}$ cover the center of another, as shown by the example in Fig 3. A new neuron $\Omega_{\gamma }^{k}=\left(m_{\gamma }^{k},c_{\gamma }^{k},S_{\gamma }^{k},U_{\gamma }^{k},w_{\gamma }^{k}\right)$ is induced to replace $\Omega_{\alpha }^{k}$ and $\Omega_{\beta }^{k}$ after merging them, and the parameters are computed and defined as follows:

$$\begin{array}{c} m_{\gamma }^{k}=m_{\alpha }^{k}+m_{\beta }^{k}, \end{array}$$ (4)

$$\begin{array}{c} c_{\gamma }^{k}=\frac{1}{m_{\gamma }^{k}}\left(m_{\alpha }^{k}x_{\alpha }^{k}+m_{\beta }^{k}c_{\beta }^{k}\right), \end{array}$$ (5)

$$\begin{array}{c} S_{\gamma }^{k}=\frac{m_{\alpha }^{k}}{m_{\gamma }^{k}}S_{\alpha }^{k}+\frac{m_{\beta }^{k}}{m_{\gamma }^{k}}S_{\beta }^{k}+\frac{m_{\alpha }^{k}m_{\beta }^{k}}{m_{\gamma }^{k}}\left(c_{\alpha }^{k}-c_{\beta }^{k}\right){\left(c_{\alpha }^{k}-c_{\beta }^{k}\right)}^{T}, \end{array}$$ (6)

$$\begin{array}{c} w_{\gamma ,l}^{k}=z_{\frac{a}{2}}\sqrt{\frac{\left|\lambda_{\gamma ,l}^{k}\right|}{m_{\gamma }^{k}}}, \end{array}$$ (7)

where $w_{\gamma ,l}^{k}\in w_{\gamma }^{k}$; $\lambda_{\gamma ,l}^{k}$ is the *l*^*th*^ eigenvalue obtained from the new covariance matrix $S_{\gamma }^{k}$; and $z_{\frac{a}{2}}$ is the *z*-value related to (1 − *a*)100% confidence interval. For Eq (7) in this work, $z_{\frac{a}{2}}=z_{0.025}=1.96$ is considered as a 95% confidence interval. After merging, both $\Omega_{\alpha }^{k}$ and $\Omega_{\beta }^{k}$ are discarded from the network. The merging process and the equations for computing all new parameters are included in the proposed learning algorithm to be discussed in the following section.

## 6 Stream Chunk Incremental Learning (SCIL) algorithm

The SCIL algorithm was proposed to handle a continuous learning scenario. A stream of class-wise data chunks is successively presented to the proposed learning algorithm. At any time, let **X**^*k*^ = {**x**_1_, **x**_2_, …, **x**_*n*_} be the set of incoming chunks of class *k*. There may be more than one class entering the learning process at any time, but SCIL learns one class at a time. The learning process consists of three algorithms. The first algorithm is the main algorithm. The second algorithm creates a new neuron and computes the parameters of the newly created neuron. The third algorithm merges two neurons and computes all parameters of the new neuron. The main process of SCIL to learn class *k* at any time is described in the following algorithm.

**SCIL Algorithm**:

**Input**: (1) Data set **X**^*k*^ of class *k* in *n*-dimensional space.

    (2) Initial width value of each created neuron.

**Output**: A set of trained neurons for data set **X**^*k*^.

1. **If** class *k* is a new class **then**

2.  Let set **Λ**^*k*^ = ∅.

3.  Create a set of hidden neurons by using **Algorithm 1** and put them in **Λ**^*k*^.

4.  Compute all parameters of neurons in **Λ**^*k*^ by using the recursive functions proposed in CIL [13].

5. **Else**

6.  **Do** lines 7-10 **Until** **X**^*k*^ is empty *or* no neuron used for updating parameter.

7.   Compute the mean vector $\bar{x}$ of the current data **X**^*k*^.

8.   Select the neuron $\Omega_{a}^{k}$ such that

$$a=\underset{1⩽s⩽d_{k}}{\text{arg}\;\text{min}}\{\psi_{s}^{k}(\bar{\text{x}})\}.$$

9.   Update all relevant parameters of $\Omega_{a}^{k}$ by using the recursive functions proposed in CIL [13].

10.   Use **Algorithm 2** to possibly merge $\Omega_{a}^{k}$ with other neurons of the same class.

11.  **EndDo**

12.  **If X**^*k*^ is not empty **then**

13.   **Do** lines 14-15 **Until X**^*k*^ is empty.

14.    Create a new hidden neuron $\Omega_{\alpha }^{k}$ by using **Algorithm 1** and set $\Lambda^{k}=\Lambda^{k}\cup \{\Omega_{\alpha }^{k}\}$.

15.    Update all relevant parameters of $\Omega_{\alpha }^{k}$ by using the recursive functions proposed in CIL [13].

16.   **EndDo**

17.  **EndIf**

18. **EndIf**

19. Discard **X**^*k*^.

**Algorithm 1**: Creating a neuron $\Omega_{\alpha }^{k}=(m_{\alpha }^{k},c_{\alpha }^{k},S_{\alpha }^{k},U_{\alpha }^{k},w_{\alpha }^{k})$

**Input**: (1) Data set **X**^*k*^ of class *k* in *n*-dimensional space.

    (2) Initial width value of each created neuron.

**Output**: A hidden neuron and updated **X**^*k*^.

1. Select randomly a data vector **x**_*i*_ ∈ **X**^*k*^.

2. Set the initial center vector $c_{\alpha }^{k}$ by $c_{\alpha }^{k}=x_{i}$.

3. Set the initial covariance matrix by $S_{\alpha }^{k}$ as a null matrix.

4. Set the orthonormal basis by $U_{\alpha }^{k}=I_{n\times n}$, where **I** is an identity matrix.

5. Set $m_{a}^{k}=1$.

6. Set each width value to an initial constant width value.

7. Set **X**^*k*^ = **X**^*k*^ − {**x**_*i*_} and discard **x**_*i*_ from the learning process.

8. Create a neuron $\Omega_{\alpha }^{k}=\left(m_{\alpha }^{k},{\bar{x}}_{\alpha }^{k},S_{\alpha }^{k},U_{\alpha }^{k},w_{\alpha }^{k}\right)$.

**Algorithm 2**: Merging two neurons in class *k*

**Input**: $\Omega_{\alpha }^{k}=\left(m_{\alpha }^{k},c_{\alpha }^{k},S_{\alpha }^{k},U_{\alpha }^{k},w_{\alpha }^{k}\right)$ and set of all neurons **Λ**^*k*^.

**Output**: Set **Λ**^*k*^ with updated content after merging some neurons.

1. Set index *β* = 1.

2. **While** ($\psi_{\beta }^{k}\left(c_{\alpha }^{k}\right)>0$ and $\psi_{\alpha }^{k}(c_{\beta }^{k})>0$) and (*β* ≠ *α*) **do**

3.   *β* = *β* + 1.

4. **EndWhile**

5. Replace neurons $\Omega_{\alpha }^{k}$ and $\Omega_{\beta }^{k}$ by $\Omega_{\gamma }^{k}$.

6. Compute the parameters $m_{\gamma }^{k},c_{\gamma }^{k},S_{\gamma }^{k}$, $w_{\gamma }^{k}$ by using (4)–(7).

7. Compute the basis vectors $U_{\gamma }^{k}$ by applying PCA to the updated matrix $S_{\gamma }^{k}$.

8. Set $\Lambda^{k}=\Lambda^{k}-\{\{\Omega_{\alpha }^{k}\}\cup \{\Omega_{\beta }^{k}\}\}$ and re-index all neurons in **Λ**^*k*^.

The time complexity *T*_*alg*_ of the Stream Chunk Incremental Learning (SCIL) algorithm is stated in the following Theorem. The proof of this theorem is given in S1 Appendix.

**Theorem 1**. *Given a data chunk having d samples with multiple classes in n*−*dimensional space, the time complexity T*_*alg*_ *of the Stream Chunk Incremental Learning (SCIL) algorithm is O*(*d*_1_*n*^2^) + *O*(*d*_2_*n*^3^), *where d*_1_ *and d*_2_ *stand for the numbers of data with new class labels and learned class labels, respectively*.

## 7 Experiments and performance evaluation

Many real-world data sets with various sizes were used to evaluate the performance of the proposed SCIL algorithm. Percentage of accuracy classification (%), the number of processing or hidden neurons, and the computational time (*s*) of the learning process are measured. The results were compared with four incremental learning methods, namely, the versatile elliptic basis function (VEBF) neural network [7], incremental learning vector quantization (ILVQ) [13], chunk incremental linear discriminant analysis (CILDA) [10], and robust incremental learning methods (RIL) [34], in which the exponential forgetting function was set to 1 for stationary class labels. All methods were implemented by MATLAB programming. Percentage of classification accuracy (%) is computed by

$$\begin{array}{c} \%\;\;accuracy=\frac{T}{N}\times 100, \end{array}$$ (8)

where *T* and *N* stand for the numbers of correct classified and all test data, respectively. Eleven real-world data sets with various sizes were examined. Ten of them are available on the University of California, Irvine [35], and the rest data set is of a physical protein-protein interaction of yeast Saccharomyces Cerevisiae [36] given in S1 Dataset. The size of each data set was determined by the product of the numbers of features and data. The attribute type of all data set is numeric. The detail of each data set is shown in Table 2. The size of each data set is computed by the product of the numbers of attributes and instances. The last column shows the ratio of the number of data in the class labels with a minimum number of data per the number of data in the class labels with a maximum number of data. The experiments were conducted on a system with Intel Core(TM) 2 Quad, 2.83 GHz processor and 6 GB RAM.

**Table 2:** Description of each data set.

Data set	Number of Attributes	Number of Instances	Size	Number of Classes	Area	Ratio of min/max
**Iris**	4	150	600	3	Life	1.00
**Yeast**	8	1,484	11,872	10	Life	0.53
**Image segmentation**	19	2,310	43,890	7	Computer	1.00
**Waveform**	21	5,000	105,000	3	Physical	1.00
**Letter recognition**	16	20,000	320,000	26	Computer	0.90
**Forest cover type**	54	581,012	31,374,648	7	Life	0.01
**Liver**	7	345	2,415	2	Life	0.73
**Spambase**	57	4,601	262,257	2	Computer	0.65
**Internet advertisement**	1,558	2,359	3,675,322	2	Computer	0.19
**Protein-protein interactions**	398	11,188	4,452,824	2	physical	1.00
**MinibooNE particle**	50	130,065	6,503,250	2	Physical	0.39

### 7.1 Experimental setting for incremental scenario

For experiments in the incremental environment, we used a 5-fold cross validation criterion to evaluate and compare the performance of SCIL and the other selected relevant methods. In 5-fold cross validation, the whole data set was randomly divided into five independent and equal-size subsets. One subset was labeled as test subset and the rest four subsets were gathered and labeled as training subset. The validation process was performed repeatedly five times so that each subset was used only once for testing. After that, the average performance is calculated among these five test subsets. To create streaming data chunks for each model, the first chunk was formed by selecting randomly 25% out of the total data in the training subset to create the initial network. For the remaining training data, *v* data points from the training set were randomly chosen to create a data chunk. A data chunk was repeatedly created until the training data set was empty. For the SCIL algorithm, each created chunk was managed into *K* class-wise data chunks, where *K* is the number of class labels in a chunk. The initial width of VEBF $w^{0}=[w_{1}^{0}\;\;w_{2}^{0}\;\;\ldots \;\;w_{n}^{0}{]}^{T}$ was computed by

$$\begin{array}{c} w_{i}^{0}=\frac{\delta }{{\left(m_{1}\right)}^{2}}\sum_{i=1}^{m_{1}}\sum_{j=1}^{m_{1}}\|x_{i}-x_{j}\|,\;\;\;i=1,\ldots ,n, \end{array}$$ (9)

where ‖⋅‖ is the Euclidean distance function, *m*_1_ is the number of data in the first chunk and *δ* is constant. Relevant parameters setting in each data set were given in Table 3. For CILDA and ILVQ, one-nearest neighbor method was used as a classifier.

**Table 3:** Parameter setting in each data set.

Data set	SCIL (*δ*)	VEBF [7] (*δ*)	ILVQ [13] (λ, *AgeOld*)
**Iris**	0.7	0.3	(21,17)
**Yeast**	0.4	1	(70,35)
**Image segmentation**	0.7	1	(180,130)
**Waveform**	0.7	1	(70,110)
**Letter recognition**	0.7	0.7	(80,100)
**Liver**	0.15	1	(16,80)
**Spambase**	0.4	1	(90,18)
**Internet advertisement**	0.7	0.7	(200,60)
**Protein-protein interaction**	0.7	1.2	(155,60)
**MinibooNE particle**	0.7	0.5	(200,150
**Forest cover type**	0.05	0.7	(280,180)

### 7.2 Experimental results

In this work, a 5-fold cross validation was used to evaluate the performance of the models. For each fold, ten of distinctive streaming data chunks in the training subset were generated. The classification accuracy, the number of hidden or processing neurons, and the computational time were measured on the testing subset shown in Tables 4, 5 and 6, respectively. For each method and each data set, the average values of the accuracy and number of neurons, computed from ten distinctive streaming chunks patterns in each of five folds, are independent and, by central limit theorem (CLT), they are normal distribution. Generally, the independent *t*-test is used to infer if there is significantly different between average values of two groups where the distribution of each group is normal and independent. So, the independent *t*-test was used to verify the statistically significant difference between the best average value and the others. Value with an asterisk (*) shows no statistical significance at a *p*-value ≥ 0.05 between the best value and the values of other methods in the same data set. The best and second best average values for each data set are identified by the bold typeface and italic typeface, respectively. Some data sets could not be learned by CILDA and RIL because of a singularity problem when solving for the weight matrix. The average rank of each method on the number of used experimental data sets is given in the last row for each set.

**Table 4:** Average classification accuracy with standard deviation $\left(\bar{x}\pm sd\right)$ of each data set.

Data set	SCIL	VEBF [7]	ILVQ [13]	CILDA [10]	RIL [34]
Iris	97.47 ± 1.45*	92.13 ± 5.92	95.73 ± 4.14*	96.17 ± 3.47*	*96.67* ± *0.00*
Image segmentation	**91.77 ± 0.80**	69.27 ± 10.52	*84.78* ± *1.76*	78.48 ± 8.66	83.74 ± 2.11
Liver	**73.33 ± 4.54**	59.77 ± 6.85	60.29 ± 5.61	62.75 ± 6.58	*63.35* ± *6.77*
Yeast	56.03 ± 2.48*	42.62 ± 12.03	49.63 ± 3.03	25.72 ± 10.77	*55.13* ± 2.90*
Letter recognition	**87.62 ± 0.42**	58.64 ± 2.33	*80.2* ± *1.17*	38.86 ± 3.33	55.51 ± 0.8
Waveform	*85.25* ± *0.75*	70.79 ± 14.19	81.71 ± 1.34	78.21 ± 1.08	**85.87 ± 0.87**
Protein-protein interaction	**89.31 ± 1.36**	50.28 ± 3.52	59.73 ± 0.67	*80.94* ± *0.54*	76.26 ± 0.59
Miniboo	*87.88* ± 0.49*	59.65 ± 11.44	86.19 ± 0.5	87.58 ± 1.36*	**90.07 ± 0.25**
Forest cover type	**80.25 ± 1.14**	63.58 ± 0.25	*73.98* ± *13.12*	51.3 ± 13.12	70.11 ± 0.15
Spambase	*90.76* ± *1.01*	68.77 ± 7.49	70.92 ± 2.44	**91.47 ± 0.83**	N/A
Internet	**95.93 ± 0.40**	64.3 ± 20.90	*89.58* ± *2.42*	N/A	N/A
**Rank average**	**1.27**	4.45	3.10	3.50	2.22

**Table 5:** Average number of used hidden neurons with the standard deviation $\left(\bar{x}\pm sd\right)$ of each data set.

Data set	SCIL	VEBF [7]	ILVQ [13]	CILDA [10]	RIL [34]
Iris	*3.76* ± *0.72*	4.28 ± 0.98	23.04 ± 9.53	120	**3**
Image segmentation	*16.96* ± *1.93*	19.68 ± 1.57	196.16 ± 56.53	1, 848	**7**
Liver	31.48 ± 5.55*	47.84 ± 4.5	*27* ± 15.62*	276	**2**
Yeast	54.56 ± 7.93	*19.08* ± *1.91*	149.36 ± 72.21	1, 187.4	**10**
Letter recognition	*30.36* ± *3.34*	235.44 ± 14.17	670.48 ± 51.47	16, 000	**26**
Waveform	*3.16* ± 0.47*	5.52 ± 2.93	177.84 ± 71.3	4, 000	**3**
Protein-protein interaction	*8.56* ± *3.08*	37.48 ± 13.43	190.2 ± 59.39	895.06	**2**
Miniboo	*78* ± *7*	2, 691 ± 423	2, 285 ± 43	104, 051.2	**2**
Forest cover type	2, 830 ± 248	*88* ± *4*	1, 550 ± 90	464, 809.6	**7**
Spambase	**13.8 ± 2.43**	*20.04* ± *1.95*	137.44 ± 27.27	3, 681.2	N/A
Internet	**7.8 ± 1.59**	*18.72* ± *2.48*	137.04 ± 47.56	N/A	N/A
**Rank average**	*2.27*	2.81	3.64	5	**1**

**Table 6:** Average computational time (*s*) with the standard deviation $\left(\bar{x}\pm sd\right)$ of each data set.

Data set	SCIL	VEBF [7]	ILVQ [13]	CILDA [10]	RIL [34]
Iris	*0.02* ± *0.004*	0.04 ± 0.004	0.07 ± 0.005	**0.003 ± 0.000**	*0.02* ± *0.002*
Image segmentation	*1.17* ± *0.01*	1.23 ± 0.08	5.77 ± 0.59	**0.03 ± 0.01**	27.26 ± 6.3
Liver	0.15 ± 0.05	0.36 ± 0.04	0.19 ± 0.03	**0.007 ± 0.009**	*0.1* ± *0.03*
Yeast	*0.23* ± *0.06*	0.54 ± 0.07	2.43 ± 0.43	**0.02 ± 0.006**	5.91 ± 1.12
Letter recognition	*0.88* ± *0.1*	18.68 ± 0.84	109.78 ± 4.61	**0.16 ± 0.02**	493 ± 145
Waveform	*0.32* ± *0.02*	2.37 ± 0.6	11.93 ± 1.76	**0.07 ± 0.01**	33.37 ± 8.7
Protein-protein interaction	*21.14* ± *3.73*	2, 266 ± 605	47.25 ± 2.13	**6.99 ± 0.38**	5, 624 ± 542
Miniboo	*65* ± *20*	936 ± 106	603 ± 59	**2.74 ± 0.08**	1, 673 ± 153
Forest cover type	202, 913 ± 60, 915	*2,451* ± *86*	38, 034 ± 465	**69 ± 6**	27, 536 ± 1, 395
Spambase	*1.24* ± *0.59*	19.93 ± 1.24	8.28 ± 0.48	**0.18 ± 0.05**	N/A
Internet	*257* ± *51*	29, 229 ± 1967	**33.5 ± 1.43**	N/A	N/A
**Rank average**	*2.36*	3.36	3.45	**1**	4.11

The accuracy for each data set is given in Table 4. The accuracy average values of SCIL are the highest in eight data sets. Significance at a *p*-value < 0.05 is found in six data sets, namely Forest cover type, Internet, Image segmentation, Letter recognition, Liver, and Protein-protein interaction, but there is no significant found in the Iris and Yeast data sets. For the Miniboo and Waveform data sets, the accuracy values of the RIL method are the highest. The accuracy of RIL is significantly greater than those of the others with *p*−value < 0.05 from the Waveform data set. For the Spambase data set, the accuracies of the SCIL and CILDA methods are slightly different. Moreover, SCIL provides the smallest values of standard deviation in most data sets. This finding implies that the influence of the incoming order of data chunks in the training process slightly affects the accuracy of the proposed SCIL when compared with the other methods. For the rank average, the SCIL method provides the best rank at 1.27.

The number of hidden neurons for each data set is given in Table 5, the average numbers of hidden neurons of CILDA and those of RIL are equal to the number of samples in the training set and the number of class labels, respectively. The average number of hidden neurons of CILDA is the worst in all the data sets. Although, the hidden neurons of RIL are the minimum value for all the data sets, the learning process cannot cope with the data of new class label. Therefore, the results of SCIL, VEBF, and ILVQ are compared. The number of hidden neurons of SCIL is significantly less than that of VEBF and ILVQ, with a *p*−value < 0.05 on eight data sets, namely, Iris, Image segmentation, Letter recognition, Waveform, Prote-in-protein interaction, MiniBooNE, Spambase, and Internet. For Liver, the number of hidden neurons of ILVQ is the lowest but is not significantly different from that of SCIL with a *p*−value ≥ 0.05. For Forest cover type and Yeast, the numbers of hidden neurons of VEBF are significantly less than those of SCIL with *p*−value < 0.05. These two numbers of hidden neurons by SCIL were obtained due to the trade-off between the number of neurons and accuracy. For classification accuracy, the standard deviation of neurons in SCIL is dramatically less than that of the other methods in almost all data sets. This finding implies that the influence of the incoming order data points in the training process does not affect the number of hidden neurons of the proposed SCIL. For the rank average, the SCIL method provides the best rank at 2.27.

For the learning time (*s*), as shown in Table 6, CILDA is the lowest in all data sets. The CILDA method consumes time only for updating the within-class scatter matrix and the between-class scatter matrix. Although the learning time of CILDA is the lowest, one of the drawbacks of CILDA is that it spends a long time assigning a class label for only the new samples that are available. This is caused by computing the distance between the new sample and each of the training data sets. The learning time of SCIL ranks second for the nine data sets, except for Liver and Forest cover type. The learning time of SCIL is slightly lower than the time of the RIL method. For Forest cover type, since the initial width of the VEBF neuron is quite small, the learning time of SCIL is quite long. However, that is the trade-off between learning time (*s*) and the accuracy of the forest cover type data set. The average rank for learning time of SCIL is 2.36.

In this work, the performance along the course test was also conducted and evaluated by *prequential* or *interleaved test-then-train* which was one of popular approaches for data stream validation [37]. The first data chunk was used to create the initial network. Then, each next data chunk was used for testing the network before it is used to incrementally update the parameters of the network. Two types of courses were considered. If a number of samples of a data set is less than 5,000, then it was divided into 11 data chunks. Otherwise, the data set was divided into 41 data chunks. For an in-depth evaluation of the performance along the course test, a paired *t*-test with a significance level of 0.05 was used to show the significant difference between the proposed SCIL and the previous CIL methods [18]. The initial width vectors of both SCIL and CIL were equal. For accuracy on the course test for each data set, the hypotheses were given by,

*H*_0_: *Accuracy of SCIL is equal to CIL along the course test*.
*H*_1_: *Accuracy on SCIL is greater than CIL along the course test*.

The test results are shown in Table 7. For Iris and Waveform, since accuracy values by SCIL and CIL are equal in every data chunk, the *p*-values of both data sets are not available, and there is no difference between SCIL and CIL on these two data sets. The accuracy of SCIL is greater than CIL along the course test on five data sets with *p*-value < 0.05. The accuracy of SCIL is equal to CIL along the course test on the left three data sets with *p*-value ≥ 0.05. The accuracy values on the last data chunk of SCIL outperform CIL on six data sets specified by the boldface number.

**Table 7:** Comparison using a paired *t*-test with a significant level of 0.05 for accuracy along the course test between SCIL and CIL methods on each data set.

Data set	Average accuracy with s.d. on the last ten chunks		Accuracy on the last chunk		*p*-value	Reject/Accept
	SCIL	CIL [18]	SCIL	CIL [18]		*H*_0_
Iris	96.88 ± 5.09	96.88 ± 5.09	95.00	95.00	N/A	−
Image segmentation	**86.38 ± 5.11**	85.24 ± 5.19	**92.86**	92.38	0.000	**Reject**
Liver	**65.61 ± 15.52**	54.45 ± 14.96	**88.57**	77.14	0.004	**Reject**
Yeast	**51.60 ± 10.10**	46.64 ± 11.14	**64.58**	59.03	0.003	**Reject**
Letter recognition	**87.99 ± 1.50**	87.62 ± 1.45	90.58	90.58	0.005	**Reject**
Waveform	85.11 ± 15.59	85.11 ± 15.59	82.50	82.50	N/A	−
Protein-protein interaction	**89.25 ± 7.82**	83.47 ± 13.05	**89.94**	74.03	0.139	*Accept*
Miniboo	97.93 ± 0.84	**98.32 ± 0.28**	**98.68**	98.52	0.000	**Reject**
Spambase	**86.34 ± 15.25**	85.59 ± 14.60	**94.06**	93.58	0.147	*Accept*
Internet	**94.39 ± 5.69**	94.19 ± 5.79	99.09	99.09	0.278	*Accept*

For the number of neurons on course test for each data set, the hypothesis test was given by

*H*_0_: *The number of neurons of SCIL is equal to that of CIL along the curse test*.
*H*1:*The number of neurons of SCIL is less than that of CIL along the course test*.

**Fig 4:**
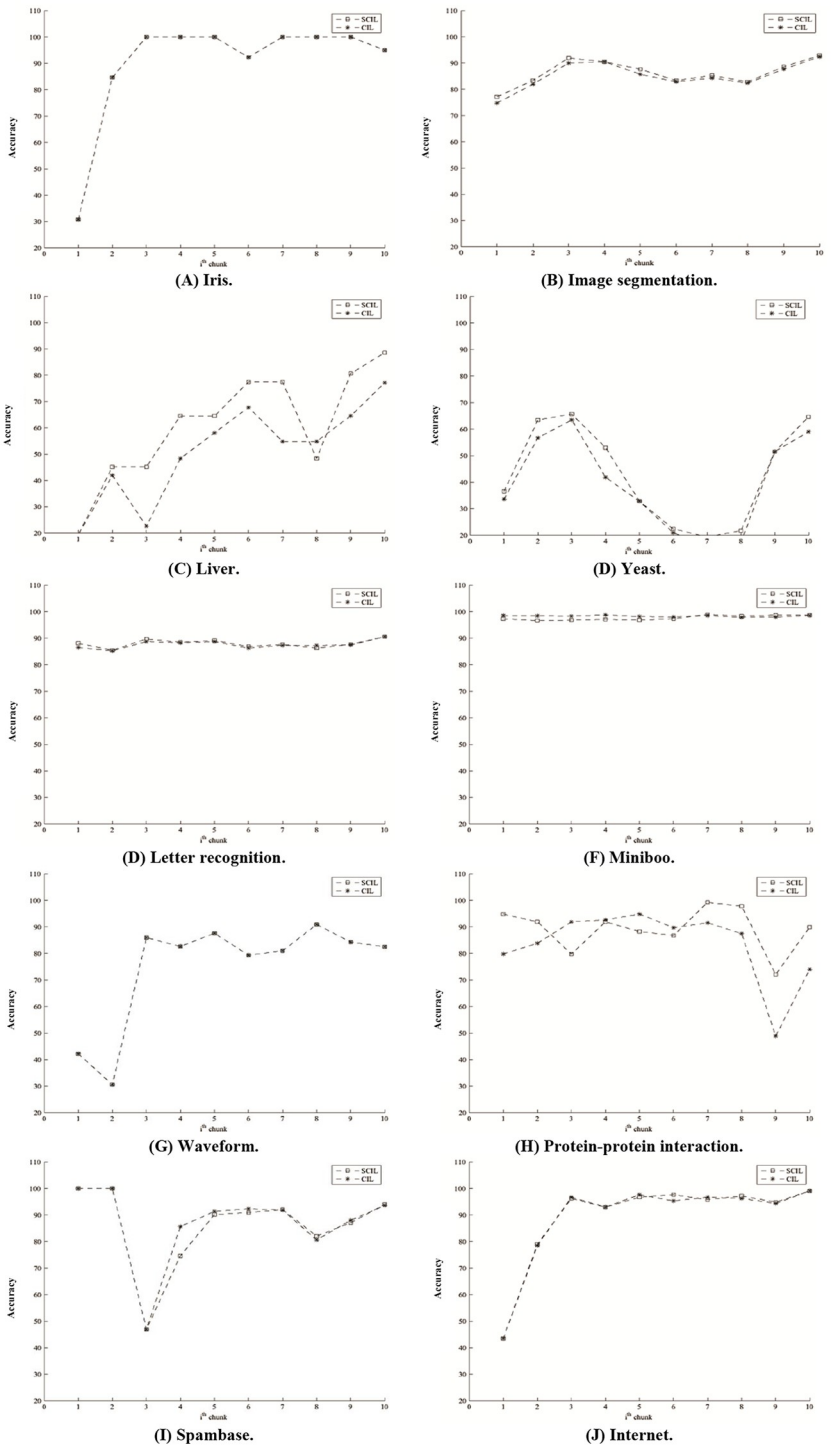
Classification accuracy on the last ten data chunks for each data set.

**Fig 5:**
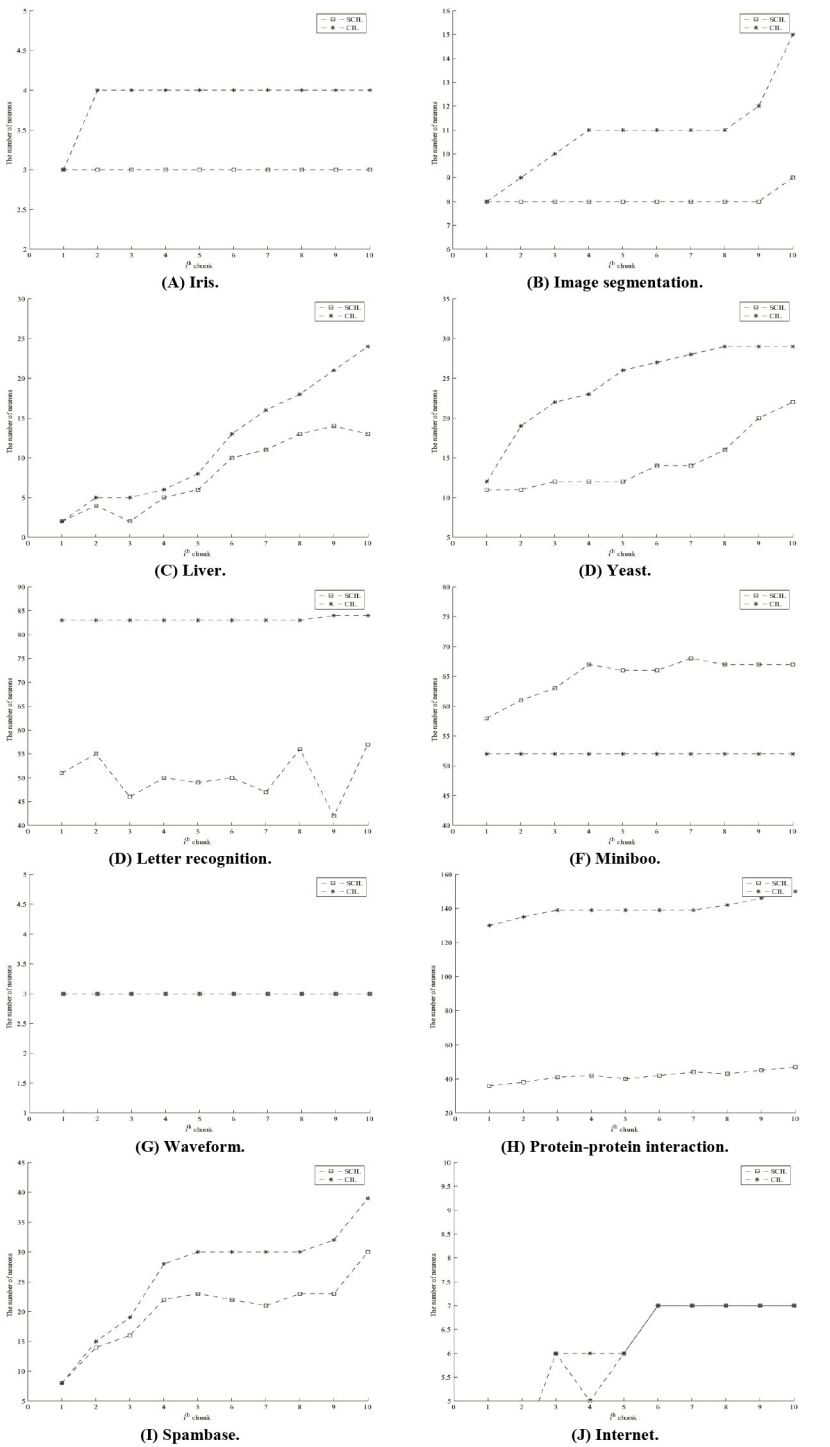
The number of hidden neurons on the last ten data chunks for each data set.

The test results are shown in Table 8. The numbers of neurons of SCIL are less than those of CIL on Iris and Waveform. The number of neurons of SCIL is less than that of CIL along course test on six data sets with *p*-value < 0.05. The number of hidden neurons on the last data chunk of SCIL is less than that of CIL on six data sets specified by the boldface number. Moreover, we observe that the average of the accuracy values on the last ten data chunks is rather high with small standard deviation values. The average of number of hidden neurons on the last ten data chunks is rather low, with respect to the number of data and with small standard deviation values, as well. Figs 4 and 5 show the average accuracy and the average number of hidden neurons on the last ten data chunks for each data set, respectively. The classification accuracy is rather stable on the last ten data chunks for eight data sets. Only liver and yeast show slightly different accuracy values, as shown in Fig 4. Additionally, the network structure could adjust itself as expressed in terms of the increase and decrease in the number of hidden neurons, as shown in Fig 5. Moreover, for comparing to the results in [17], they applied random forest with stratified random sampling and Bloom filtering for Forest Cover Type data set. The average accuracy(%) along the course test of the propose SCIL method is 89.62 which is quite higher than those of [17] for both filtered data from actual data and from sampled data which were 76.62 and 75.58, respectively.

**Table 8:** Comparison using a paired *t*-test with a significance level of 0.05 for the number of used neurons along the course test between SCIL and CIL methods on each data set.

Data set	Average number of neurons on the last ten chunks		Number of neurons on the last chunk		*p*-value	Reject/Accept
	SCIL	CIL [18]	SCIL	CIL [18]		*H*_0_
Iris	**3.00 ± 0.00**	4.00 ± 0.00	**3**	4	N/A	−
Image segmentation	**8.11 ± 0.31**	11.22 ± 1.55	**9**	15	0.000	**Reject**
Liver	**8.67 ± 4.22**	12.89 ± 6.84	**13**	24	0.002	**Reject**
Yeast	**14.78 ± 3.64**	25.78 ± 3.42	**22**	29	0.000	**Reject**
Letter recognition	**50.3 ± 4.47**	83.20 ± 0.40	**57**	84	0.000	**Reject**
Waveform	3.00 ± 0.00	3.00 ± 0.00	3	3	N/A	−
Protein-protein interaction	**41.8 ± 3.09**	139.80 ± 5.19	**47**	150	0.000	**Reject**
Miniboo	65.00 ± 3.10	**52 ± 0.00**	67	**52**	0.374	*Accept*
Spambase	**21.60 ± 4.29**	28.11 ± 6.69	**30**	39	0.000	**Reject**
Internet	**6.22 ± 1.03**	6.33 ± 0.94	7	7	0.278	*Accept*

## 8 Conclusion

This paper presented the Stream Class-wise Incremental Learning (SCIL) algorithm for a versatile elliptic basis function neural network (VEBFNN) to handle the stream of data chunks. In this study, each incoming chunk contains multiple classes. One important aspect of the proposed learning algorithm is based on the discard-after-learn concept. The created network can adapt itself to learn new knowledge without forgetting old one, which is opposite to that of the stability-plasticity dilemma. For performance evaluation, accuracy (%) and number of used neurons of SCIL are measured and compared to the four incremental methods. The experimental results showed that the accuracy of SCIL are higher than that of the others for most data sets. Only Waveform and Miniboo data sets, the accuracy of SCIL is lightly less than that of the RIL method. In addition, the number of neurons of the SCIL is less than those of the VEBF, ILVQ, CILDA and CIL methods for most data sets. For RIL, the number of hidden neurons is determined by the number of class labels. For Forest cover type, SCIL uses more number of neurons than VEBF because of the trade-off between accuracy and the number of neurons. For the learning time, the learning time of CILDA is the lowest for all data sets, but CILDA takes an extremely long time to assign a class label for a new sample. The learning time of SCIL is the second lowest for nine data sets, as shown in underlined numbers, except for liver and forest cover type. The learning time of SCIL is slightly lower than the time of the RIL method. For Forest cover type, since the initial width of SCIL is quite small, the time of SCIL is quite high, which is the trade-off between learning time and accuracy for forest cover type. Moreover, the proposed method is capable of increasing or decreasing the number of hidden neurons, according to the widths based on the *z*-score with a 95% confidence interval. Thus, the over fitting problem due to the excessive number of neurons can be easily diminished.

All experiments were conducted by using a single processing unit. However, it is possible to deploy the capability of graphic card to speed up the computational time, especially step 8 in the main **SCIL Algorithm** and steps 2-4 in **Algorithm 2**, since these steps have no data dependency among them.

## Supporting information

S1 Appendix Proof of Theorem 1. (PDF)

S1 Dataset A physical protein-protein interaction of yeast Saccharomyces Cerevisiae data set. (RAR)
