# Supplementary material for: Streaming chunk incremental learning for class-wise data stream classification with fast learning speed and low structural complexity
Source: PLoS One. 2019 Sep 9;14(9):e0220624. doi: 10.1371/journal.pone.0220624 (PMC6733468; doi:10.1371/journal.pone.0220624)
Supplement: S1 Appendix — (PDF) [file pone.0220624.s001.pdf]

Theorem 1. Given a data chunk having d samples with multiple classes in n-dimensional space, the time complexity T_{alg} of the Stream Chunk Incremental Learning (SCIL) algorithm $O(d_1n^2) + O(d_2n^3)$, where d_1 and d_2 stand for the numbers of data with new class labels and learned class labels, respectively.

Proof. There are three situations to be considered. The first situation is the learning time for the first data chunks. The second situation is the learning time for data chunks without any new class label. The last situation is the learning time when there is a mixture of already learned classes and some new class labels. The analysis of the learning time for each situation is as follows. Let a_k be the amount data in class k.

In the first situation, the learning steps are steps 1-4. Suppose all class labels are in set $\mathbf{K}$. The number of created neurons in each class by **Algorithm 1** is equal to $\sum_{k\in\mathbf{K}} a_k = d$. There are five parameters in each neuron and n dimensions. All parameters concern the number of dimensions. Hence, the time spent by **Algorithm 1** is equal to $O(5n\sum_{k\in\mathbf{K}} a_k) = O(5nd) = O(dn)$. After creating neurons, the values of all parameters are computed in step 4. For each neuron, the time to compute m_α^k is $O(n)$, center $\mathbf{c}_\alpha^k$ is $O(n)$, covariance $\mathbf{S}_\alpha^k$ is $O(n^2)$, bases $\mathbf{U}_\alpha^k$ is $O(n^2)$, and widths $\mathbf{w}_\alpha^k$ is $O(n)$. Therefore, the total time complexity to compute all parameters of all data in step 4 is $O(dn) + O(dn) + O(dn^2) + O(dn^2) + O(dn^2) = O(dn^2)$.

In the second situation, most of the time spent is at step 10, which is the execution of **Algorithm 2**. In **Algorithm 2**, finding the overlapped neurons in the same class k at steps 2-4 takes $O(a_kn)$. Computing the parameters at steps 6-7 takes $O(a_k)$ for the mean, $O(a_kn^2)$ for covariance matrix, and $O(a_kn^3)$ for eigenvectors and eigenvalues. Thus, the total time complexity for class k is $a_kn^2 + a_kn^3$. For all classes, the time complexity is $\sum_{k\in\mathbf{K}}(a_kn^2 + a_kn^3) = O(dn^3)$.

In the last situation, let d_1 be the number of data with new class labels, and d_2 is the number of data with learned class labels. Obviously, $d_1 + d_2 = d$. The time complexity to learn the data with new class labels is $O(d_1n^2)$, and the time complexity to merge neurons of the data with learned class labels is $O(d_2n^3)$. Therefore, the total time complexity is $O(d_1n^2) + O(d_2n^3)$. □
